# Polyethylene-Based Phase Change Materials Modified with Hexagonal Boron Nitride Nanoparticles with Enhanced Thermal Stability and Thermal Conductivity

**DOI:** 10.3390/ma19030455

**Published:** 2026-01-23

**Authors:** Beata Macherzyńska, Adrianna Pitera, Katarzyna Nowicka-Dunal, Kinga Pielichowska

**Affiliations:** Department of Glass Technology and Amorphous Coatings, Faculty of Materials Science and Ceramics, AGH University of Krakow, Al. Mickiewicza 30, 30-059 Krakow, Poland; beatam@agh.edu.pl (B.M.); apitera@student.agh.edu.pl (A.P.); nowicka@agh.edu.pl (K.N.-D.)

**Keywords:** phase change materials, polyethylene wax, boron nitride, thermal energy storage

## Abstract

Polyethylene waxes (PEWs) are considered promising mid-temperature phase change materials (PCMs). However, their low thermal conductivity limits both applicability and efficiency. One of the more interesting inorganic additives for PCMs is boron nitride (BN), which exhibits high thermal conductivity while remaining electrically insulating, excellent chemical and thermal stability, and good oxidation resistance. In this study, PEW was modified with hexagonal boron nitride (h-BN) in the range of 0.025 to 0.5 wt.%. Differential scanning calorimetry (DSC) results revealed that the addition of h-BN significantly alters the phase-transition behavior of polyethylene wax, broadens the melting and solidification temperature ranges, and reduces supercooling from 11 °C to 9 °C. Thermogravimetric analysis (TGA) showed that the incorporation of h-BN improves the thermal stability of the material. The temperature corresponding to 5% mass loss increased by about 50 °C after incorporation of more than 0.025% h-BN. The temperature of maximum mass-loss rate (T_DTGmax_) was shifted about 8 °C toward higher temperatures. FTIR results indicate that h-BN does not change the chemical structure of polyethylene waxes, but does affect their morphology and physical properties by increasing the thermal conductivity from 0.30 to 0.40 mW/K. These effects enable the design of composites with tunable properties for energy-storage applications.

## 1. Introduction

Phase change materials (PCMs) represent an attractive group of materials for effective thermal energy storage in a wide range of applications, from construction to industry. Their ability to absorb and release large amounts of heat during phase transitions, such as melting or solidification, is effectively utilized. Solid–liquid phase change materials are the most commonly used type of heat accumulators. They release energy in the form of heat during the transition from liquid to solid, and then store a comparable amount of energy during melting [1,2]. The heat storage process involves three stages: heating the material to its phase-transition temperature, the phase transition itself, and further heating above the transition point. This enables heat to be stored in two forms—sensible heat and latent heat.

Unlike inorganic materials, which exhibit relatively high thermal conductivity but also several limitations, such as a tendency to supercool, phase separation, or corrosion, organic PCMs offer a wider range of phase change temperatures, comparable latent heat values, and improved thermal stability [3,4]. Among the most commonly used organic compounds in PCMs are paraffins, valued for their low cost, broad range of phase change temperatures (from −180 to 130 °C), and high latent heat of phase change (180–230 J/g) [5]. However, despite their numerous advantages, the use of paraffins in pure form is limited by several significant factors, which necessitate additional modification to optimize the material’s performance and durability.

A particularly interesting organic phase change material is polyethylene wax. It is an ethylene oligomer with a low average molecular weight, typically below 10,000 [6]. The methods to obtain polyethylene wax can be divided into three main categories: direct synthesis from monomer, thermal degradation of polyethylene (pyrolysis), and separation of wax as a byproduct during polyethylene production [7].

Polyethylene wax is a fully synthetic, polyolefin-based wax. Over the past fifty years, the production and consumption of plastics have increased significantly, directly contributing to growing environmental pollution. Current recycling methods indicate that pyrolysis is one of the most economically effective strategies for plastic waste management. Polyethylene wax obtained through pyrolysis has traditionally been considered a relatively low-value raw material, used primarily for the production of chemicals and fuels, as an asphalt additive, or for further refining in petrochemical plants.

Over the past two years, research has emerged suggesting the potential use of polyethylene wax as a medium temperature phase change material for thermal energy storage. To date, studies have focused on modifying paraffins and paraffin waxes by incorporating polyethylene wax to improve the thermal properties of these systems. Polyethylene waxes contain hydrocarbon chains ranging from 5 to 69 carbon atoms, with the solid fraction—comprising molecules containing more than 18 carbon atoms—being the dominant component, accompanied by a small admixture of lighter, liquid paraffin hydrocarbons. [8].

Due to its thermal properties, polyethylene wax is a promising candidate for medium-temperature phase-change materials. Studies have shown that both high- and low-density polyethylene waxes, depending on the production method, exhibit relatively high heats of fusion comparable to those of paraffin waxes, ranging from 120 to 210 J/g [9]. The melting points of these materials range from 84 to 128 °C [10], depending on the polymer chain length. Furthermore, polyethylene waxes demonstrate good thermal stability both at the phase-transition temperature and in multiple thermal cycles [8].

Previous research indicates that, due to their specific thermal and chemical properties, polyethylene waxes offer significant potential for modification, which could lead to improved performance and expanded application possibilities [11].

To improve the thermal conductivity of phase change materials, several common modification methods have been explored in the literature. An effective strategy is the addition of high thermal conductivity nanoparticles to pure PCMs, which significantly enhances their thermal properties and overall performance [12]. Among various modifiers, such as silicon oxide, graphite, and carbon nanotubes, hexagonal boron nitride (h-BN) stands out due to its exceptional in-plane thermal conductivity (300–2000 W/m·K) [13], which far exceeds its out-of-plane thermal conductivity (approximately 30 W/m·K) [14,15]. Moreover, h-BN exhibits excellent electrical insulation as well as high chemical and thermal stability, particularly under extreme conditions such as elevated temperatures and strongly acidic or alkaline environments [16].

The structure of hexagonal boron nitride resembles that of graphite, with layers of boron and nitrogen atoms replacing carbon layers, which has led to its colloquial name, “white graphite,” due to its characteristic white color. Owing to this structure, h-BN exhibits not only excellent physical properties but also remarkable thermal stability, resisting oxidation up to approximately 1000 °C in air, 1400 °C in vacuum, and 2800 °C in an inert atmosphere. The material is also non-toxic and chemically inert [17]. These unique properties make hexagonal boron nitride a highly promising additive to enhance the thermal properties and general functionality of phase-change materials [18]. Incorporation of h-BN may significantly contribute to the development of innovative energy technologies and improve the efficiency of industrial processes [19].

The aim of this study was to analyze the effect of hexagonal boron nitride on the thermal properties of organic phase change materials and to evaluate their potential applications in thermal energy storage systems. The influence of the additive on parameters such as phase-change heat, degree of supercooling, thermal stability, and degree of crystallinity was investigated. Furthermore, the quality of nanoparticle dispersion within the material was assessed.

## 2. Materials and Methods

Phase change materials were prepared using CERALENE^®^ 2E (EuroCeras, Kędzierzyn-Koźle, Poland) low-density polyethylene wax, characterized by an average molecular weight of 2500–3500, and hexagonal boron nitride (h-BN) from PlasmaChem GmbH, Berlin Germany, with a purity exceeding 98.5% and an average particle size of 500 ± 100 nm. Polyethylene wax (PEW) samples were prepared by adding a specified percentage of h-BN to the wax, with the masses of the individual components precisely measured to achieve the desired additive content in the final 10 g samples. All ingredients were weighed using a KERN ABT 220-4M (Sigma-Aldrich, St. Louis, MO, USA) analytical balance with an accuracy of ±0.0001 g and placed in polypropylene containers. A summary of the phase change materials obtained and the corresponding h-BN percentages is provided in Table 1.

The samples were then thermally treated in a Jeio Tech 665L Vacuum Oven (OV-12) (Medline Scientific, Rotherham, UK) at 130 °C for 2 h to completely melt the polyethylene wax. The melted samples were then homogenized using a SONICS Vibra-Cell (Sonics & Materials, Inc., Newtown, CT, USA) ultrasonicator for 30 s, after which the materials were allowed to solidify at room temperature.

A Mettler Toledo DSC1 differential scanning calorimeter was used to measure the thermal properties of the samples. Measurements were performed at a constant heating rate of 10 °C/min and a cooling rate of –10 °C/min under a nitrogen atmosphere (flow rate: 30 mL/min). Samples of approximately 4 mg were sealed in perforated aluminum crucibles, with an empty aluminum crucible used as the reference. To eliminate the influence of thermal history, results from the second heating cycle were analyzed. The degree of subcooling was determined using Equation (1):

(1) $$∆T=T_{m}-T_{c}[{}^{\circ}C]$$

where *T_m_* is the maximum melting point, and *T_c_* is the maximum solidification temperature. The degree of crystallinity (*X_c_*) was determined using the Formula (2):

(2) $$X_{c}=\frac{\Delta H_{m}}{(1-w_{BN})\cdot \Delta H_{m,100\%}}\times 100\%$$

where Δ*H_m_* denotes the melting enthalpy of the sample, *w_BN_* is the mass fraction of the hexagonal boron nitride addition, and Δ*H_m_*_,100%_ = 293 J/g represents the melting enthalpy of fully crystalline polyethylene [20].

Temperature-modulated DSC measurements were performed using a Mettler Toledo DSC 1 (Mettler Toledo, Greifensee, Switzerland) calorimeter equipped with the TOPEM DSC option. Prior to the measurements, all samples were melted at a heating rate of 20 °C/min and a cooling rate of −20 °C/min. The subsequent measurements were carried out at a heating and cooling rate of 2 °C/min, with a modulation amplitude of ±0.5 °C and a pulse width ranging from 15 to 36 s.

Thermogravimetric analysis (TGA) was performed using a TA Instruments TGA 550 Discovery analyzer (TA Instruments, New Castle, DE, USA). Samples with mass ca. 3 mg were placed in open platinum crucibles and heated from 35 to 600 °C at a constant rate of 10 °C/min under a nitrogen atmosphere (flow rate: 50 mL/min).

FTIR spectra were obtained using a Thermo Scientific Nicolet Apex FTIR spectrophotometer (Thermo Scientific, Waltham, MA, USA) with the ATR method on a diamond crystal, in the range of 4000 to 600 cm^−1^ at a resolution of 4 cm^−1^. Thermal conductivity measurements were performed using the laser flash analysis (LFA) method with a Netzsch LFA 427 analyzer (Netzsch, Selb, Germany). The samples were flat disks, and the measurements were carried out at a temperature of 20 ± 2 °C. During the test, the lower surface of the sample was heated with a short laser pulse, and the resulting temperature changes on the upper surface were recorded with an infrared detector. Based on these data, the instrument determined the thermal diffusivity coefficient (α, mm^2^/s), which was then used to calculate the thermal conductivity (λ) according to the following equation:

λ (T) = ρ(T)⋅*C*_p_(T)⋅α(T) (3)

where λ—thermal conductivity [W/m·K], ρ—density [g/cm^3^], *C*ₚ—specific heat at constant pressure [J/g·K], and α—thermal diffusivity [mm^2^/s].

**Figure 1:**
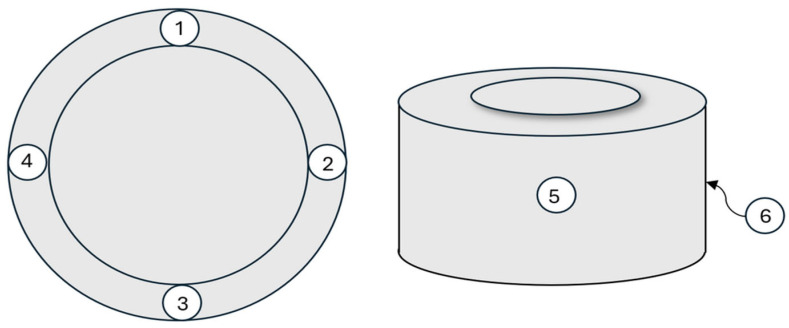
Showing the points where ultrasonic wave velocity measurements were taken.

To assess the degree of homogenization and dispersion of the hexagonal boron nitride additive, non-destructive ultrasonic testing was conducted using a Materials Tester CT3 (Unipan Ultrasonic, Mettler Toledo, Greifensee, Switzerland), featuring an ultrasonic wave transit-time accuracy of ±0.01 μs. The testing procedure involved measuring the propagation velocity of longitudinal waves in samples containing the modifier and comparing the results with those obtained for a reference sample. Ultrasound at a frequency of 1 MHz was used to determine the ultrasonic velocities of all PEW samples. Measurements were taken across each sample’s thickness (points 1–4 in Figure 1) and diameter (points 5–6 in Figure 1). The heterogeneity (H) and anisotropy (A) of the samples were also determined according to the following formulas:

(4) $$H=\frac{C_{max\;}-C_{min\;}}{C_{max\;}}\cdot 100\;\;[\%]$$

where *C_max_*—maximum longitudinal wave propagation velocity in a given direction, *C_min_*—minimum longitudinal wave propagation velocity in a given direction:

(5) $$A=\frac{C_{D\;}-C_{T\;}}{C_{D\;}}\cdot 100\;\;[\%]$$

where *C_D_*—average longitudinal wave propagation velocity along the diameter, *C_T_*—average longitudinal wave propagation velocity along the thickness.

## 3. Results

**Figure 2:**
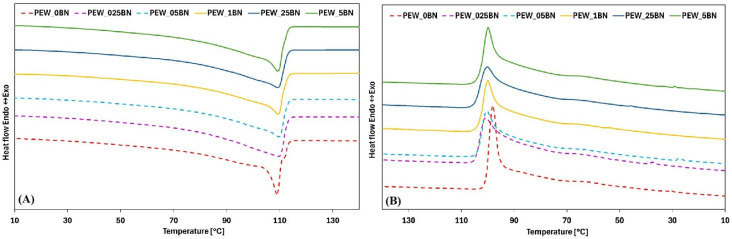
DSC curves of PEW with various h-BN contents for a melting (second heating cycle) (**A**), and solidification (**B**).

Waxes are mixtures of molecules of varying size and nature and therefore do not have a specific melting point, as do pure substances [21]. Therefore, during DSC testing of PE waxes, the onset, maximum, and end temperatures of the transformation were recorded. The DSC analysis results are presented in Figure 2A,B, while Table 2 and Table 3 summarize thermal parameters for individual samples, such as the onset and end temperatures of the phase transition, the maximum transition temperature, the enthalpy of the transformation, and the degree of supercooling and crystallinity.

The DSC curves of paraffin waxes reported in the literature clearly show that PEWs exhibit two characteristic features: a large peak corresponding to crystal melting and a smaller peak associated with a solid–solid transition [22]. In Figure 2A, this behavior is clearly visible for the unmodified PEW_0BN polyethylene wax. A large peak was observed at approximately 106 °C. Another small transition was detected between 55 and 65 °C for the PE wax samples. These values are consistent with the data reported by Yetgin et al. [22], although they show slight deviations from the thermal property results obtained for certain PE waxes used in industrial applications [23]. Three fractions can be distinguished: at 50 °C, a peak corresponding to the wax fraction with the lowest molecular weight; at approximately 60 °C, a peak associated with a fraction of moderately low molecular weight; and a final peak occurring at 90–100 °C, attributed to the melting of the low-density polyethylene present in the sample [22].

Analysis of the effect of hexagonal boron nitride on the melting curves (Figure 2A) indicates that the addition of h-BN produces only minor changes in their overall shape. The reference sample, without any additive, is characterized by a sharp rise and a narrow peak, whereas the samples containing h-BN exhibit broader peaks. For samples with addition levels of 0.025% to 0.05% by weight, a flattening of the curves is observed, and the phase-transition temperature range widens from 8 °C (reference sample) to 45 °C (0.025% h-BN). As the additive content increases, this range decreases, reaching 28 °C for 0.25% and further narrowing to 15 °C for the 0.5% sample. A reduction in the heat of fusion is also observed, which is consistent with previous reports from the literature. The initial heat of fusion of the reference sample was 154 J/g, decreasing with increasing additive content, showing a trend above 0.1% but not returning to the initial value.

Similar changes in the solidification curves (Figure 2B) confirm the melting-curve observations. The reference sample exhibits a sharp decline and a narrow peak, while the h-BN-modified samples display broader peaks and a shift in the onset of crystallization to approximately 104 °C. Additionally, a small exothermic peak is present between 40 and 20 °C for samples with low addition levels (0%, 0.025%, 0.05%, and 0.5%). The crystallization curves are flatter in the presence of h-BN, with solidification temperature ranges of 6 °C (reference) and 15 °C (0.025% h-BN), with variable values at higher addition levels.

The observed decrease in heat release indicates a reduction in transformation enthalpy, which is particularly pronounced for samples containing 0.05% and 0.25% additives. It is also worth noting the minimal differences in the enthalpy of phase transformation between the melting and solidification processes for the individual samples.

The reduction in phase-transition enthalpy resulting from the addition of hexagonal boron nitride is attributed to its high thermal conductivity, which promotes faster heat dissipation within the material. In both transformations analyzed, the increase in the transformation temperature range correlates with a reduction in the degree of supercooling—11 °C for the reference sample and 8 °C for the sample containing 0.025% additive, respectively. The degree of crystallinity initially decreases to 45% at an additive content of 0.1%. With further increases in nanoadditive concentration, the degree of crystallinity rises again, although it does not return to the level of the reference sample. The initial decrease is explained by the disruption of the crystalline structure due to nanoparticles becoming incorporated between the wax chains, while the subsequent increase above 0.1% is associated with the nanoadditive acting as crystallization nuclei, thereby promoting crystal growth around the particles.

As shown in Table 2, the addition of h-BN initially reduces latent heat (heat of melting and heat of solidification), but after exceeding the minimum for the addition of 0.01%, it slowly increases. This is closely related to the change in crystallinity of the samples.

The observed decrease in latent heat indicates a reduction in transformation enthalpy, which is particularly pronounced for samples containing 0.05% and 0.25% additives. It is also worth noting the minimal differences in the enthalpy of phase transitions between the melting and solidification processes for the individual samples. The reduction in phase-transition enthalpy resulting from the addition of hexagonal boron nitride is attributed to its high thermal conductivity, which promotes faster heat dissipation within the material, and it is connected to the effect of h-BN on the PEW crystallization process and degree of crystallinity.

It is hypothesized that, in the early stages of crystallization, polymer spherulites nucleate more rapidly in the presence of nanoparticles. However, during later stages, the presence of nanoparticles can create spatial obstruction, leading to reduced crystallinity [24]. Biswas et al. [25] also observed that crystallization of polymer chains from the melt may cause additive aggregation outside the crystalline regions.

**Figure 3:**
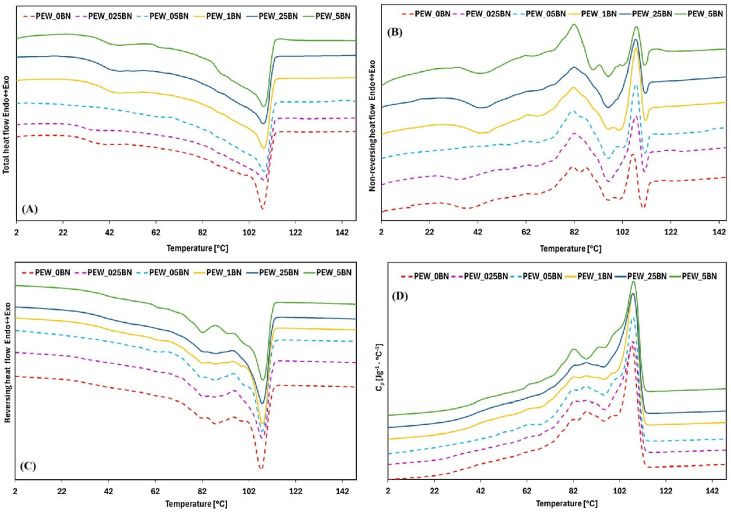
TOPEM DSC curves after heat flux deconvolution into the irreversible component (**A**), into the reversible component (**B**). TOPEM DSC total heat flux curves (**C**), heat capacity curve (**D**).

The use of modulated differential scanning calorimetry (TOPEM^®^ DSC) enabled a more precise melting analysis of the tested samples. The results obtained using this technique are presented in Figure 3. In all analyzed samples, the melting process was observed to occur at a temperature several degrees lower than that obtained using the conventional DSC method, a typical phenomenon resulting from the slower heating rate typical of modulated techniques.

Analysis of the total heat flux curves revealed the presence of double peaks, with minima in the temperature ranges of 35–55 °C and 105–110 °C, with the exception of the sample containing 0.05% by mass of hexagonal boron nitride (BN). The first temperature minimum was broader for the samples containing 0.1% and 0.25% BN. For most BN-modified samples, a more gradual decline in the curve was observed compared with the reference sample. In contrast, the second minimum was characterized by a sharp decline only in the reference sample, whereas the curves for the BN-containing materials exhibited greater smoothness.

In the analysis of the reversible component of the DSC signal, numerous endothermic and exothermic effects were detected, with their temperatures shifting toward higher values as the BN content increased. A reduction in the number of these effects was observed for the sample with 0.25% BN, while the samples containing 0.5% BN showed the most distinct curve patterns.

The most pronounced changes were observed in the irreversible component, where the addition of BN broadened the endothermic peaks and increased their amplitude. Samples with a BN content above 0.05% displayed a broader first endothermic peak, with the minimum shifted toward higher temperatures. Additionally, all BN-modified samples showed a reduction in one exothermic effect that was visible at approximately 80 °C in the reference sample. The second exothermic effect exhibited a lower peak for the sample with 0.5% BN, while the peak maximum of all BN-containing samples was shifted toward higher temperatures relative to the reference. The final endothermic effect showed a smaller decrease in all modified samples compared to the unmodified sample.

Analysis of heat capacity changes as a function of temperature demonstrated that the samples with 0.075% and 0.5% BN were characterized by a narrower exothermic effect at approximately 82.5 °C, while the remaining samples showed a broader effect and a gentler curve profile. Moreover, for the above-mentioned BN-modified samples, endothermic effects were recorded at approximately 85 °C and 90 °C, which disappeared at 95 °C, whereas in the remaining samples these effects were clearly visible.

The endothermic effect at 103 °C, observed for the reference sample, shifted toward higher temperatures in all samples with BN additives, except those containing 0.025% and 0.25% BN, for which this effect disappeared. The exothermic effect at 107 °C for the unmodified sample shifted several degrees toward higher temperatures in all modified samples, and the samples with 0.05%, 0.1%, and 0.25% BN showed greater intensity of this effect compared with the reference sample.

**Figure 4:**
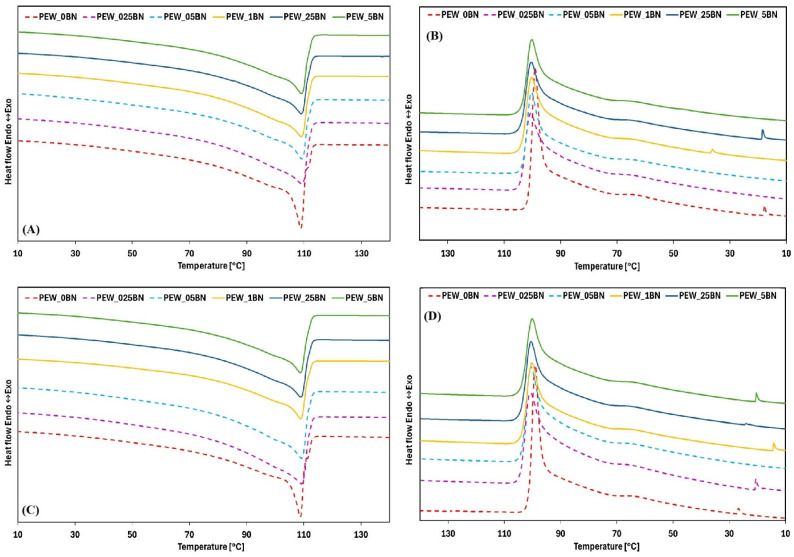
DSC curves for PEW with different h-BN contents after 50 melting and solidification cycles: in air (**A**) melting and (**B**) solidification; in nitrogen (**C**) melting and (**D**) solidification.

The results of the DSC analysis after 50 melting and solidification cycles, performed in air and nitrogen, are presented in Figure 4A–D. Table 4 and Table 5 summarize the thermal parameters for each sample, including the onset and end temperatures of the phase transition, the maximum transition temperature, and the enthalpy of the transition. Table 6 presents the calculated values of supercooling and crystallinity.

Analysis of the effect of 50 melting cycles on pure PEW and PEW modified with h-BN, presented in Figure 4A,C (in air and nitrogen, respectively), indicates minimal changes in the overall shape of the melting curves. The reference sample, without any additive, is characterized by a sharp rise and a narrow peak, whereas the samples containing h-BN exhibit broader peaks. For samples with an additive content of 0.025 wt.% or more, a flattening of the curves is observed, and the phase-transition temperature range expands from 9 °C (reference sample) to 25 °C and 27 °C in air and nitrogen, respectively. With increasing additive content, this range decreases, reaching 20 °C and 18 °C at 0.5% addition. These values differ slightly from the results shown in Figure 2A.

Changes in the heat of fusion were observed and were comparable across all samples, regardless of h-BN content, with slightly lower values recorded for samples subjected to cycles in a nitrogen atmosphere. However, the heat of fusion decreased significantly relative to the initial tests (Table 2). For the reference sample, the heat of fusion decreased by 23% and 28% in air and nitrogen, respectively. For the modified samples, the nanoadditive contributed to a reduction in the heat of fusion of less than 20%, with a decreasing trend as the h-BN content increased.

The decrease in heat release indicates a reduction in the enthalpy of the phase transition. It is also worth noting the minimal differences in enthalpy values between the melting and solidification processes for individual samples. The reduction in enthalpy after repeated melting and solidification cycles is attributed to the degradation of polyethylene wax. This is confirmed by the decrease in crystallinity from 53% to 37% for the reference sample and from approximately 46% to 37% for the h-BN-modified samples.

Changes in the solidification curves (Figure 4B,D) demonstrated very similar behavior for all samples, regardless of h-BN content. The only notable difference was a shift in the onset of solidification to approximately 104 °C. Additionally, a small exothermic peak was observed between 40 °C and 10°C. The crystallization curves were sharper and occurred over a narrower temperature range than those shown in Figure 2B, with solidification temperature ranges of 15 °C for the reference sample, 11 °C for samples cycled in air, and 10 °C for samples cycled in nitrogen. For the reference sample, the phase-transition range after 50 cycles widened from 6 °C to 15 °C, irrespective of the atmosphere.

Comparison of the DSC results before and after 50 cycles shows that the melting and solidification temperatures did not change, consistent with observations reported by other authors [23,24]. However, the decrease in heat release confirms the reduction in phase-transition enthalpy. As noted above, this reduction is attributed to polyethylene wax degradation and is supported by the corresponding decrease in crystallinity.

**Figure 5:**
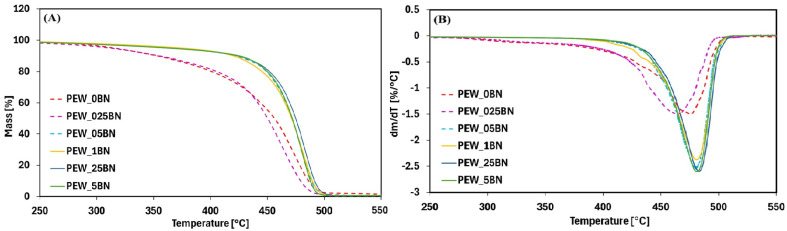
TG curves (**A**), DTG curves (**B**) of the tested samples.

The results of the thermogravimetric analysis are presented in Figure 5 and Table 7.

TG analysis showed that the addition of hexagonal boron nitride (h-BN) above 0.025 wt% had a beneficial effect on the thermal stability of polyethylene wax. The TG and DTG curves are presented in Figure 5, and Table 7 summarizes the temperatures corresponding to selected weight losses (1%, 5%, 10%, 50%), the temperature of the maximum weight-loss rate (T_DTGmax_), and the amount of solid residue at 600 °C.

A substantial increase in thermal stability was observed with increasing h-BN content, particularly at 5% and 10% weight losses. Thermal stability at 5% weight loss increased by 45 °C (for 0.25% addition) and by 58 °C (for 0.1%), while at 10% loss, it increased from 67 °C (0.1%) to 72 °C (0.5% addition). This enhanced stability can be attributed to the high thermal conductivity of h-BN, which promotes efficient heat dissipation and effectively limits thermal degradation. A smaller but noticeable stability increase (~2 °C) was also recorded for the 0.025% addition at 10% weight loss.

At 1% weight loss, only the sample with 0.1% h-BN showed an improvement, with a 3 °C increase in stability. Thermal stability at 50% weight loss increased to a lesser extent than at 5% and 10% loss, rising from 15 °C (0.05% addition) to 19 °C (0.25%).

The addition of h-BN also shifted the T_DTGmax_—the temperature of maximum mass-loss rate—toward higher values, from 4 °C (0.025% addition) to 8 °C (0.5% addition). However, for the 0.025% sample, this temperature was shifted 12 °C lower, likely indicating insufficient additive content.

The solid residue after decomposition at 600 °C was approximately 1% for the reference sample and decreased with increasing additive content, reaching 0.4–0.7%. It is worth noting that the initial mass of the reference sample was nearly twice that of the 0.025% sample, while the 0.05% sample had a higher initial mass than the reference, but still only about half as much. The higher residue of the reference sample may suggest the formation of less volatile decomposition products, which requires confirmation by infrared spectroscopy. For samples containing 0.1% h-BN or more, the residue remained at 0.6–0.7% [22].

In all samples containing h-BN, the residue at 600 °C was lower than in the reference sample, potentially due to the presence of the nanoadditive and differences in sample crystallinity. Modification of polyethylene wax with hexagonal boron nitride significantly affects the crystalline structure, which likely translates into different thermochemical behavior at elevated temperatures. The increased thermal stability associated with h-BN has been reported in the literature, with an emphasis on the high thermal conductivity of h-BN, which enhances heat dissipation and suppresses thermal degradation [17].

The shifts in maximum decomposition-rate temperatures and the reduction in residual mass at 600 °C can be attributed to structural modifications induced by h-BN, which have been confirmed to improve the thermal resistance of nanocomposites.

Controlled dosing of hexagonal boron nitride influences both the thermal and structural properties of polyethylene wax, consistent with reports that demonstrate improved stability and durability of materials used under demanding thermal conditions [17].

**Figure 6:**
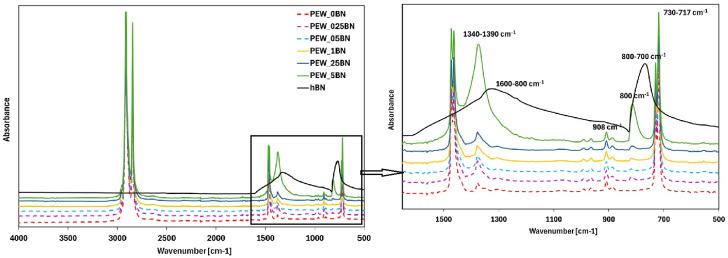
FTIR spectra for polyethylene wax without and modified with h-BN addition.

The results of the FTIR test are presented in Figure 6.

The absorption band located at 2915 cm^−1^ is attributed to stretching vibrations of the CH_2_ group, characteristic of the amorphous phase, while the band near 2847 cm^−1^ corresponds to symmetric stretching vibrations of the same bonds, present in both the amorphous and crystalline phases. Absorption bands associated with deformation vibrations of C–H bonds were detected in the 1450–1480 cm^−1^ range. Additionally, low-intensity bands in the 1340–1390 cm^−1^ range, resulting from deformation vibrations of CH_3_ groups, were observed [26,27,28,29]. With increasing hexagonal boron nitride content, an intensification and broadening of the absorption band in the 1340–1390 cm^−1^ range is observed, along with a decrease in the intensity of the bands at 1467–1462 cm^−1^. The band at 908 cm^−1^ is ambiguous and may represent stretching vibrations of a mono-substituted aromatic ring or deformation vibrations of the C–H bond. Given the absence of aromatic structures in PE wax, this band is attributed to torsional vibrations of the C–H bond. The bands in the 730–717 cm^−1^ region correspond to rotational vibrations within the CH_2_ group, while additional vibrational modes appear between 705 and 735 cm^−1^. In samples with the highest h-BN content (0.05%), a band near 800 cm^−1^ was identified, corresponding to bending vibrations of B–N–B bonds, while the band at 1385 cm^−1^ originated from stretching vibrations of B–N bonds in the h-BN plane, confirming the presence of hexagonal boron nitride nanoparticles in the PE/h-BN wax nanocomposite. The analyses confirmed the presence of functional groups typical of LDPE, including symmetric and asymmetric C–H stretching vibrations, angular deformations of CH_2_ and CH_3_ groups, and rotational deformations of CH_2_. These results are consistent with data from the literature, allowing samples to be classified as low-density polyethylene [24].

Polymers such as PE or PE waxes that possess in their structure only C-C covalent bonds and C-H bonds, without any polar groups, have limited noncovalent interactions (e.g., van der Waals or hydrogen bonding) with BN nanosheets [30]. As can be seen in Figure 6, no additional absorption bands were detected in FTIR spectra, which confirmed that the chemical structure of PCM remained stable [31]. It was found that the infrared absorption bands of the PEW with h-BN mixed in the molten state by sonication were nearly identical to those of the unmodified PEW. Moreover, characteristic absorption bands for this system were found at very similar wavenumbers.

In general, incorporation of BN enhances phonon transport through the formation of conduction pathways. Another possibility is the role of h-BN nanoparticles as nucleating agents for PEW crystallization. However, in PCM, the crucial point is to keep the heat of a phase transition at a high level, and increasing BN content will decrease the heat of phase transition.

**Figure 7:**
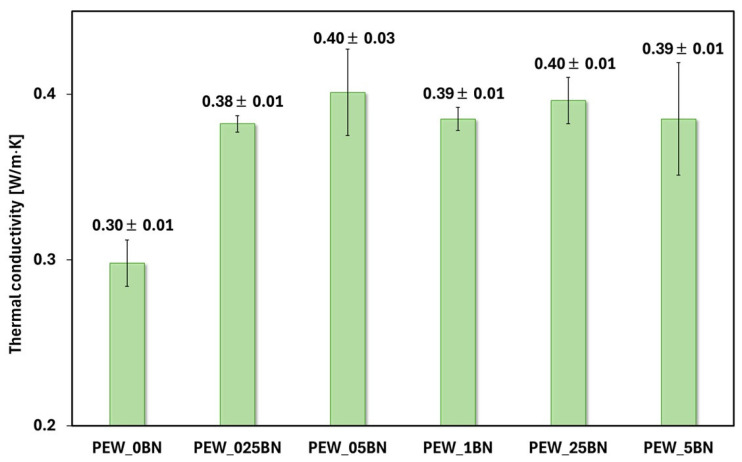
Thermal conductivity vs. the amount of h-BN in PCM nanocomposites.

The effect of BN on the thermal conductivity of PE waxes was investigated using the LFA method, and the results are presented in Figure 7.

**Figure 8:**
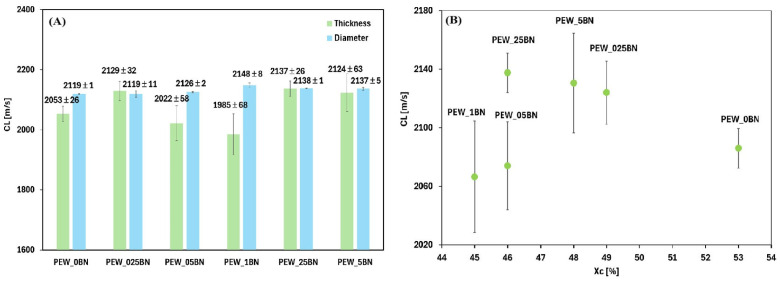
Dependence of the propagation velocity on the amount of h-BN addition (**A**), on the degree of crystallinity (**B**).

The reference sample (PEW_0BN) exhibits the lowest thermal conductivity, so polyethylene wax itself is a poor thermal conductor compared to the PEW/h-BN nanocomposites. Increasing the content of hexagonal boron nitride in the polyethylene wax leads to a significant increase in the thermal conductivity compared to the reference sample without the additive, which is consistent with data described in the literature [19,32]. The highest thermal conductivity values are observed for composites with a small addition of h-BN (PEW_025BN and PEW_05BN), suggesting the existence of a favorable concentration range within which an effective heat conduction path is created in the polymer matrix. With further increases in the boron nitride content (PEW_1BN, PEW_25BN, PEW_5BN), the thermal conductivity remains elevated compared to pure wax but no longer increases significantly, which may be due to saturation of the system or the appearance of agglomerates that limit conduction efficiency. It should be noted that the error bars are relatively small, indicating good measurement repeatability and stable thermal properties of the obtained materials. For the range above 0.025%, the addition of h-BN does not cause large variations in conductivity between samples—the values are similar, so a lower concentration of the nanoadditive (e.g., 0.05%) can be selected and achieve a similar effect to a higher amount, reducing cost and the risk of agglomeration. The obtained longitudinal ultrasonic wave propagation velocities are shown in Figure 8A for measurements along the thickness and diameter. Figure 8B shows the dependence of the velocity on the degree of crystallinity.

Measurements across the sample’s thickness were taken at the sample edges, which were in contact with the container walls—this location could have influenced the polymer solidification process and the nanoadditive concentration. The greatest discrepancy in measurements occurred for the sample with 0.1% h-BN, where the propagation velocity of the longitudinal ultrasonic wave ranged from 1700 to 2200 m/s, which could be due to the uneven distribution of the nanoadditive within the sample. However, measurements across the diameter demonstrated the greatest consistency of the results. Analysis of the relationship between the obtained results and the degree of crystallinity indicates that materials with a higher degree of crystallinity often exhibit better structural cohesion, which may favor higher ultrasonic wave propagation velocity. However, the obtained data do not allow for unambiguous confirmation of this correlation, as the highest longitudinal ultrasonic wave propagation velocity was not observed for the reference sample PEW_0BN, with the highest degree of crystallinity. Low-intensity ultrasound propagation in polymers, acting as high-frequency dynamic mechanical deformation, corresponds to changes in the elastic properties of polymers associated with glass transition, crystallization, cross-linking, and other chemical and physical phenomena associated with changes in viscoelastic properties. The velocity of ultrasonic waves is related to the storage modulus and density of the polymer, while the absorption of ultrasonic waves is related to energy dissipation in the material and, therefore, to the loss modulus [33,34,35]. Analysis of the results along the diameter indicates an initial decrease and then an increase in the propagation velocity, which, in addition to the effect of the degree of crystallinity, may be related to changes in the internal structure of the material. The addition of boron nitride increases the anisotropy and heterogeneity of the samples (Table 8). These results suggest both agglomeration of the nanoadditive and its uneven distribution within the sample volume.

## 4. Conclusions

In this work, mid-temperature PCMs based on polyethylene waxes with enhanced thermal conductivity have been obtained. The effect of h-BN on the latent heat of phase transition, degree of supercooling, thermal stability, degree of crystallinity and thermal conductivity was investigated. Furthermore, the quality of nanoparticle dispersion within the material was assessed. The obtained results revealed that

- The addition of hexagonal boron nitride (h-BN) significantly modifies the course of phase transformations in polyethylene wax: it acts as an effective heterogeneous nucleation nucleator, extends the melting and solidification temperature range, reduces supercooling, and leads to a competition between facilitated nucleation and limited crystal growth.
- Melting/solidification cycles cause thermal degradation, primarily through the observed decrease in the heat of phase transition and the degree of crystallinity.
- The introduction of h-BN improves the thermal stability of the material (higher mass loss temperatures and T_DTGmax_ and lower decomposition residues) but simultaneously reduces the enthalpy of phase transformations, which indicates the difficulty in forming large, well-ordered crystals, leading to the formation of more defect-prone crystal structures.
- The results of DSC, TGA, FTIR, thermal conductivity, and ultrasound measurements are consistent with each other: they show that hBN does not change the chemical structure of polyethylene waxes but its morphology and physical properties—it increases thermal conductivity, modifies the degree of crystallinity, and local ordering, which allows the design of composites with adjustable properties for applications in heat storage and operation at elevated temperatures.

## Figures and Tables

**Table 1:** List of sample names and amounts of h-BN added.

Name of Samples	h-BN Addition [Mass %]
PEW_0	0
PEW_025	0.025
PEW_05	0.05
PEW_1	0.1
PEW_25	0.25
PEW_5	0.5

**Table 2:** Temperatures and heats of phase-transitions PEW with different h-BN content.

Samples	T_m onset_ [°C]	T_m_ [°C]	T_m endset_ [°C]	Heat of Phase Transitions [J/g]
Melting				
PEW_0	103	109	111	154
PEW_025	68	109	113	145
PEW_05	88	110	113	135
PEW_1	95	109	112	131
PEW_25	85	109	113	134
PEW_5	97	109	112	140
Solidification				
PEW_0	101	98	95	154
PEW_025	104	101	89	145
PEW_05	104	101	92	134
PEW_1	104	100	94	132
PEW_25	105	100	90	134
PEW_5	104	100	95	141

**Table 3:** DSC analysis results of PEW with different h-BN content.

Samples	ΔT [°C]	X_c_ [%]
PEW_0	11	53
PEW_025	8	49
PEW_05	9	46
PEW_1	9	45
PEW_25	9	46
PEW_5	9	48

**Table 4:** Temperatures and heats of phase-transitions PEW with different h-BN content after 50 melting and solidification cycles in air.

Samples	T_m onset_ [°C]	T_m_ [°C]	T_m endset_ [°C]	Heat of Phase Transitions [J/g]
Melting				
PEW_0	102	109	111	119
PEW_025	87	109	112	117
PEW_05	95	109	112	118
PEW_1	91	109	112	119
PEW_25	92	109	112	125
PEW_5	94	109	112	119
Solidification				
PEW_0	101	99	96	107
PEW_025	104	101	92	104
PEW_05	103	101	94	109
PEW_1	104	101	93	101
PEW_25	104	101	93	107
PEW_5	104	100	94	109

**Table 5:** Temperatures and heats of phase-transitions PEW with different h-BN content after 50 melting and solidification cycles in nitrogen.

Samples	T_m onset_ [°C]	T_m_ [°C]	T_m endset_ [°C]	Heat of Phase Transitions [J/g]
Melting				
PEW_0	102	109	111	111
PEW_025	85	109	112	113
PEW_05	91	109	112	117
PEW_1	94	109	112	127
PEW_25	92	109	112	107
PEW_5	93	109	112	129
Solidification				
PEW_0	101	99	96	110
PEW_025	104	101	92	101
PEW_05	104	101	94	98
PEW_1	104	100	94	107
PEW_25	104	101	94	101
PEW_5	104	100	93	104

**Table 6:** DSC analysis results of PEW with different h-BN content after 50 melting and solidification cycles.

Samples	In Air		In Nitrogen	
	ΔT [°C]	X_c_ [%]	ΔT [°C]	X_c_ [%]
PEW_0	10	37	10	38
PEW_025	8	36	8	34
PEW_05	8	37	8	33
PEW_1	8	35	9	37
PEW_25	8	37	8	35
PEW_5	9	37	9	36

**Table 7:** Thermal stability of the tested materials.

Samples	Mas [mg]	T_1%_ [°C]	T_5%_ [°C]	T_10%_ [°C]	T_50%_ [°C]	T_max DTG_ [°C]	Residue at 600 °C [%]
PEW_0	2.52	247	315	353	456	476	1.0
PEW_025	1.54	223	312	354	450	464	0.4
PEW_05	2.66	240	364	422	472	480	0.4
PEW_1	4.20	250	373	420	471	481	0.6
PEW_25	3.07	235	360	423	472	483	0.7
PEW_5	3.63	233	363	425	472	481	0.6

**Table 8:** Heterogeneity and anisotropy of the samples calculated from ultrasonic measurements.

Samples	Heterogeneity [%]	Anisotropy [%]
PEW_0	3.12	3.11
PEW_025	3.23	0.49
PEW_05	6.66	4.86
PEW_1	6.89	6.76
PEW_25	21.44	7.60
PEW_5	2.58	0.05

## Data Availability

The original contributions presented in this study are included in the article. Further inquiries can be directed to the corresponding author.
